# Balanced Distribution Adaptation for Metal Oxide Semiconductor Gas Sensor Array Drift Compensation

**DOI:** 10.3390/s21103403

**Published:** 2021-05-13

**Authors:** Zongze Jiang, Peng Xu, Yongbin Du, Feng Yuan, Kai Song

**Affiliations:** 1 School of Instrumentation Science and Engineering, Harbin Institute of Technology, Harbin 150001, China; jiangzz@hit.edu.cn (Z.J.); duyongbin@hit.edu.cn (Y.D.); 2 Command and Control Engineering College, People’s Liberation Army Engineering University, Nanjing 210007, China; ljgcdxxp@163.com; 3 School of Electrical Engineering and Automation, Harbin Institute of Technology, Harbin 150001, China

**Keywords:** drift compensation, balanced distribution adaptation, transfer learning, feature extraction, sensor array, domain adaption

## Abstract

Drift compensation is an important issue for metal oxide semiconductor (MOS) gas sensor arrays. General machine learning methods require constant calibration and a large amount of label gas data. At the same time, recalibration will cause a lot of costs, and label gas is difficult to obtain in practice. In this paper, a novel drift compensation method based on balanced distribution adaptation (BDA) is proposed. First, the BDA drift compensation method can adjust the conditional distribution and marginal distribution between the two domains through the weight balance factor, thereby more effectively reducing the mismatch between the two domains. When the BDA method performs classification tasks through machine learning, no labeled data is required in the target domain. Then, the particle swarm optimization algorithm is used to improve the accuracy of drift compensation. Individuals in the population are initialized randomly, and their fitness values are calculated. Iterative optimization of the population individuals is conducted until the optimal weight balance factor parameters are calculated. Finally, the BDA method is experimentally verified on the public gas sensor drift data set. Experimental results showed that the BDA method was significantly better than the existing joint distribution adaptation (JDA) method and other standard drift compensation methods such as K-Nearest Neighbor (KNN). In the two setting groups, the recognition accuracy was 4.54% and 1.62% ahead of the JDA method, and 12.23% and 15.83% ahead of the KNN method.

## 1. Introduction

With the advantages of small size, low cost, simple production, and high sensitivity to flammable and toxic gases, metal oxide semiconductor (MOS) sensor arrays play a vital role in the fields of environmental protection and monitoring [1,2], food and beverage production [3,4], clinical diagnosis, and process control [5,6]. MOS sensor arrays also are currently the most commonly used information acquisition devices in machine olfactory systems [7,8,9,10]. When the mixed gas enters the gas chamber, the oxygen ions adsorbed on the surface of the MOS gas sensors will chemically react with them, which will cause the resistance of the MOS gas sensors to decrease sharply. Then, pattern-recognition technology is applied to process these signals to identify the composition of the mixed gas and estimate its concentration. However, in this sensor, the drift effect is also obvious. The drift phenomenon causes the sensor input and output relationship to change. Even for the same type and concentration of the measured gas, the sensor output values measured at different times before and after are different. When the sensor drifts, the sensor input and output relationship obtained in the calibration phase will be destroyed. The results of gas classification are difficult to obtain accurately. Therefore, it is necessary to find an effective method to detect and compensate the drift of gas sensor.

The drift compensation research of MOS gas sensor arrays can be traced back to the 1990s, but it is still a thorny issue [11,12]. The drift compensation methods for gas sensors can be roughly divided into the following three categories: (1) signal preprocessing methods, (2) composition correction methods, and (3) adaptive correction methods. In the first categories of approaches, a baseline processing method and frequency domain filtering method are frequently used. These two methods compensate for the output response of each sensor in the array. But due to the complexity of the drift cause, these methods can only reduce part of the adverse effects caused by the drift. The component correction methods attempt to find and remove drift-sensitive components before the model is built. Due to insufficient prior information, such methods are usually unable to effectively deal with drifting samples that differ greatly from the initial distribution. In practice, component correction methods require researchers to recalibrate the sensor system frequently. It usually takes several weeks to retrain the classifier network of the sensor array with new pure calibration samples of the labeled gas for each type of gas that the sensor array can recognize. However, retraining the classifier network is time-consuming, and it is also difficult to regularly obtain pure calibration samples of new gases.

Adaptive correction methods were first applied to drift compensation by [13] through classifier integration in 2012. The experimental results proved that the ensemble method based on support vector machines can deal with sensor drift well and perform better than the baseline competing method. Zhang et al. [14] proposed a domain-adaptive extreme learning machine (DAELM) drift compensation method based on semisupervised learning. This method obtained a higher classification accuracy, but also required more labeled drift samples to participate in the model construction. Liu et al. [15] used stacked autoencoders and restricted Boltzmann machines to introduce deep learning into sensor drift compensation. Luo et al. [16] showed a deep belief network (DBN) to preprocess gas sensor data. The coupling between each characteristic of the sensor data was enhanced by this method. It helped the depth features of the data to be effectively extracted and expressed. In addition, the combination of this method and support vector machine (SVM) was effective through numerical experiments. Yan et al. [17] realized drift compensation by finding the invariance between the original sample domain and the drift sample domain. This method added the background information of the two sample domains to the original features of the sample through feature enhancement preprocessing. Liu et al. [18] developed a domain transfer broad learning system (DTBLS) based on BLS. The DTBLS framework uses labeled source data and unlabeled target data to learn a robust target classifier to adaptively compensate for drift in sensor response. Liu et al. [19] established an adaptive domain-based subspace learning method. This method considered both maximizing tag feature correlation and minimizing feature redundancy (DMDMR) to solve the drift problem based on the gas sensor arrays. The proposed method learned time-varying common subspace with similar distributions for regular and recently drifted gas sensor arrays responses to adapt to the inconsistent data distribution caused by drift. Leon-Medina et al. [20] proposed a transfer learning method to solve the drift problem by using the joint distributed adaptive (JDA) method, which could adapt to the marginal and conditional distribution between domains.

The above adaptive correction methods applied in drift compensation of MOS gas sensor arrays has achieved certain effects. However, most methods based on neural networks require a large amount of label data in the target domain. In order to address this issue, we learned from the above methods, and propose a novel drift compensation model based on BDA in this paper. There is no need to label data in the target domain, and it can improve the recognition accuracy and robustness of the sensor array for a long time. Furthermore, unlike the JDA method, which directly ignores the importance of the two, the BDA method uses a weight balance factor to evaluate the importance of each distribution. Therefore, the BDA algorithm has higher accuracy. In actual measurement, the BDA method needs to be combined with a preprocessing device, gas measurement cell, air pump, solenoid valve, and thermostat control unit in the system. These units make the temperature, humidity, air flow, and other parameters of the sample gas closer to the laboratory environment, which helps the accuracy of drift compensation results.

The structure of this paper is organized as follows. In Section 2, the related work of BDA and particle swarm optimization (PSO) method, a brief description of the combination of transfer learning, and MOS sensor array drift compensation are introduced. Section 3 describes the drift compensation methodology with all its parts. In Section 4, the effect of the BDA algorithm is verified on the public data set and compared with the state-of-the-art transfer learning method to demonstrate the effectiveness of the method. Finally, the conclusions are drawn in Section 5.

## 2. Theoretical Background

### 2.1. Transfer Learning

**Figure 1:**
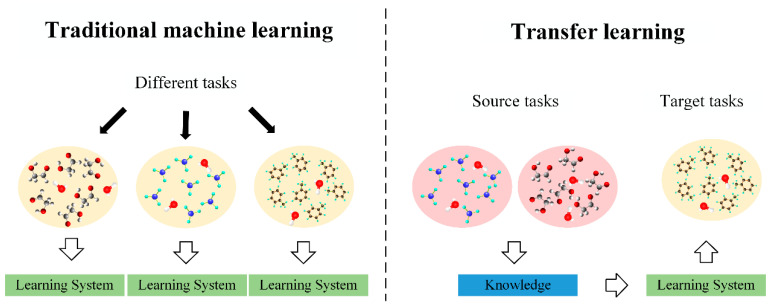
The differences between the transfer learning approach and the traditional machine learning approach.

Transfer learning is used to transfer the knowledge learned in the source domain to the target domain to help the target domain carry out model training [21,22]. Domain and task are the two basic concepts of transfer learning. Figure 1 shows the differences between the transfer learning approach and the traditional machine learning approach.

Transfer learning can also be expressed more accurately by domains and tasks.

**Definition** **1.** *Given a labeled source domain* $D_{s}={\left\{x_{i},y_{i}\right\}}_{i=1}^{n}$*and an unlabeled target domain*$D_{t}={\left\{x_{j}\right\}}_{j=n+1}^{n+m}$, *the data distributions P*(*x_s_*) *and P*(*x_t_*) *of these two fields are different. The purpose of transfer learning is to use the knowledge of D_s_ to learn the knowledge (label) of the target domain D_t_*.

When drift occurs, the characteristic distribution of the target domain *D_t_* (with drift) data will not obey the source domain *D_s_* (without drift). The generalization ability of the classifier is reduced due to drift, which leads to the performance degradation of the classifier trained with the labeled data of *D_s_* when tested on *D_t_*. Obviously, the category space between the two domains affected by drift is the same; that is, *Y_s_* = *Y_t_*. However, the marginal distributions of these two domains are different (*P*(*x_s_*) ≠ *P*(*x_t_*)), and the conditional probability distributions are also different (*P*(*y_s_|x_s_*) ≠ *P*(*y_t_|x_t_*)). The goal of transfer learning is to use labeled data *D_s_* to train a classifier *f*: *x_t_* **→** *y_t_* to predict the label *y_t_* ∊ *Y_t_* of the target domain *D_t_*_._

### 2.2. Balanced Distribution Adaptation

**Figure 2:**
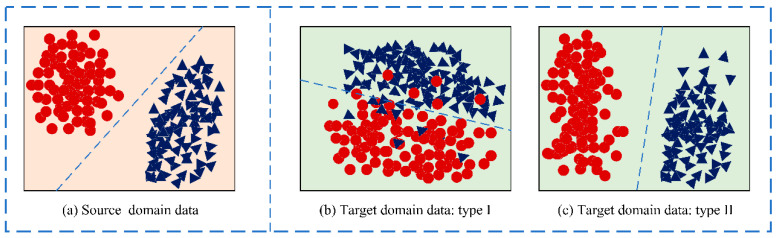
Target domain data with different data distribution.

Distribution adaptation is one of the most commonly used transfer learning methods. The basic idea of this method is that the data probability distribution of the source domain and the target domain are different. Then, the most direct way is to narrow the distance between different data distributions through some transformations. Figure 2 visually shows several data distribution situations. Simply put, the difference in the edge distribution of the data means that the data is not similar overall. The conditional distribution of the data is different; that is, the overall data is similar, but specific to each category, they are not very similar.

A major problem that needs to be solved in transfer learning is to reduce the distribution difference between the source domain and the target domain. JDA assumes that marginal distribution and conditional distribution are equally important and adapted.

(1) $$\begin{array}{cc} D(D_{s},D_{t}) & \approx D\left(P(x_{s}),P(x_{t})\right)\\ & +D\left(P(y_{s}|x_{s}),P(y_{t}|x_{t})\right) \end{array}$$

However, for different situations, the marginal distribution and the conditional distribution play different roles, which takes us back to the problem of the two distributions shown in Figure 2. Obviously, when the target domain is the situation shown in Figure 2b, the marginal distribution should be given priority; when the target domain is the situation shown in Figure 2c, the conditional distribution should be given priority. JDA and subsequent extension work ignored this issue. Therefore, the weight balance factor *μ* is added to the BDA to leverage the importance of each distribution:

(2) $$\begin{array}{cc} D(D_{s},D_{t}) & \approx (1-\mu )D\left(P(x_{s}),P(x_{t})\right)\\ & +\mu D\left(P(y_{s}|x_{s}),P(y_{t}|x_{t})\right) \end{array}$$

where *μ* ∈ [0,1]. When *μ* → 0, it means that there is a large difference between the source domain data set and the target domain data set, so the marginal distribution is more dominant; when *μ* → 1, it reveals the data sets between domains have high similarity, so conditional distribution adaptation is more important to adapt. When *μ* = 0.5, BDA degenerates to JDA. In other words, the weight balance factor *μ* can adjust the importance of each distribution to obtain good results.

We adopted the maximum mean discrepancy (MMD) to minimize the distance of marginal distribution *P*(*x_s_*), *P*(*x_t_*) and conditional distribution *P*(*y_s_|x_s_*), *P*(*y_t_*|*x_t_*) between the source domain and the target domain, which is:

(3) $$\begin{array}{cc} D(D_{s},D_{t}) & \approx (1-\mu ){\left\|\frac{1}{n}\sum_{i=1}^{n}x_{si}-\frac{1}{m}\sum_{j=1}^{m}x_{tj}\right\|}_{H}^{2}\\ & +\mu \sum_{c=1}^{c}{\left\|\frac{1}{n_{c}}\sum_{x_{s_{i}}\subset D_{s}}^{n}x_{si}-\frac{1}{m_{c}}\sum_{x_{t_{j}}\subset D_{t}}^{m}x_{tj}\right\|}_{H}^{2} \end{array}$$

where *H* denotes the reproducing kernel Hilbert space (RKHS); *c* ∈ {1,2,…,*C*} represents various class labels; n and m represent the number of samples in the source domain and target domain, respectively; and *D_s_* and *D_t_* denote the samples belonging to class c in the source domain and target domain, respectively. *n_c_* = |*D_s_*|, *m_c_* = |*D_t_*| is the number of samples in *D_s_* and *D_t_*. These two items represent the marginal distribution distance and conditional distribution distance between the source domain and the target domain, respectively.

Further application of matrix tricks and regularization to Equation (2) results in:

(4) $$\begin{array}{l} \min\;\mathrm{tr}\left(A^{T}X\left(\left(1-\mu \right)M_{0}+\mu \sum_{c=1}^{C}M_{c}\right)X^{T}A\right)+\lambda {\left\|A\right\|}_{F}^{2}\\ s.t.\;A^{T}XHX^{T}A=I,\;0\leq \mu \leq 1 \end{array}$$

Equation (4) contains two terms: the first term represents the adaptation of the balance factor of the marginal distribution and the conditional distribution, and the second item is the regularization term. There are two constraints included in Equation (4). The first constraint is to preserve the inner properties of the transformed data (**A**^T^**X**) consistent with the original data. The second constraint limits the balance factor *μ* to the range, where the input data matrix **X** is composed of ***x****_s_* and ***x****_t_*.

Furthermore, **A** represents the transformation matrix. **I** represents the identity matrix, and **I** ∈ R^(^*^n^*^+^*^m^*^)×(^*^n^*^+^*^m^*^)^. **H** is the centered matrix, which can be expressed specifically as **H** = **I** − (1/n)1. **M**_0_ and **M***_c_* are the matrices belonging to the MMD matrix, which can be constructed in the following ways:

(5) $${\left(M_{0}\right)}_{ij}=\{\begin{array}{l} \frac{1}{n^{2}},x_{i},x_{j}\in D_{s}\\ \frac{1}{m^{2}},x_{i},x_{j}\in D_{t}\\ \frac{-1}{mn},\mathrm{otherwise} \end{array}$$

(6) $${\left(M_{c}\right)}_{ij}=\{\begin{array}{l} \frac{1}{n_{c}{}^{2}},x_{i},x_{j}\in D_{s}^{\left(c\right)}\\ \frac{1}{m_{c}{}^{2}},x_{i},x_{j}\in D_{t}^{\left(c\right)}\\ \frac{-1}{m_{c}n_{c}},\{\begin{array}{c} x_{i}\in D_{s}^{\left(c\right)},x_{j}\in D_{t}^{\left(c\right)}\\ x_{i}\in D_{t}^{\left(c\right)},x_{j}\in D_{s}^{\left(c\right)} \end{array}\\ 0,\mathrm{otherwise} \end{array}$$

**Learning Algorithm:** The Lagrange multiplier is denoted as Φ = (*ϕ*1,*ϕ*2,…,*ϕd*), then the Lagrange function for Equation (4) can be expressed in the following form:

(7) $$\begin{array}{cc} L & =\mathrm{tr}\left(A^{T}X\left(\left(1-\mu \right)M_{0}+\mu \sum_{c=1}^{C}M_{c}\right)X^{T}A\right)\\ & +\lambda {\left\|A\right\|}_{F}^{2}+tr\left(\left(I-A^{T}XHX^{T}A\right)\Phi \right) \end{array}$$

where set derivative ∂*L*/∂**A** = 0. Then, the optimization of the Equation (7) can be converted into a generalized eigendecomposition problem to derive:

(8) $$\left(X\left(\left(1-\mu \right)M_{0}+\mu \sum_{c=1}^{C}M_{c}\right)X^{T}\right)+\lambda I=XHX^{T}A\Phi$$

Finally, by solving Equation (8), we can obtain the optimal transformation matrix **A** and its *d* smallest eigenvectors at the same time.

The value of *μ* must be estimated based on the data distribution. We evaluated its performance through the drifted classification accuracy value in the experiment.

**Figure 3:**
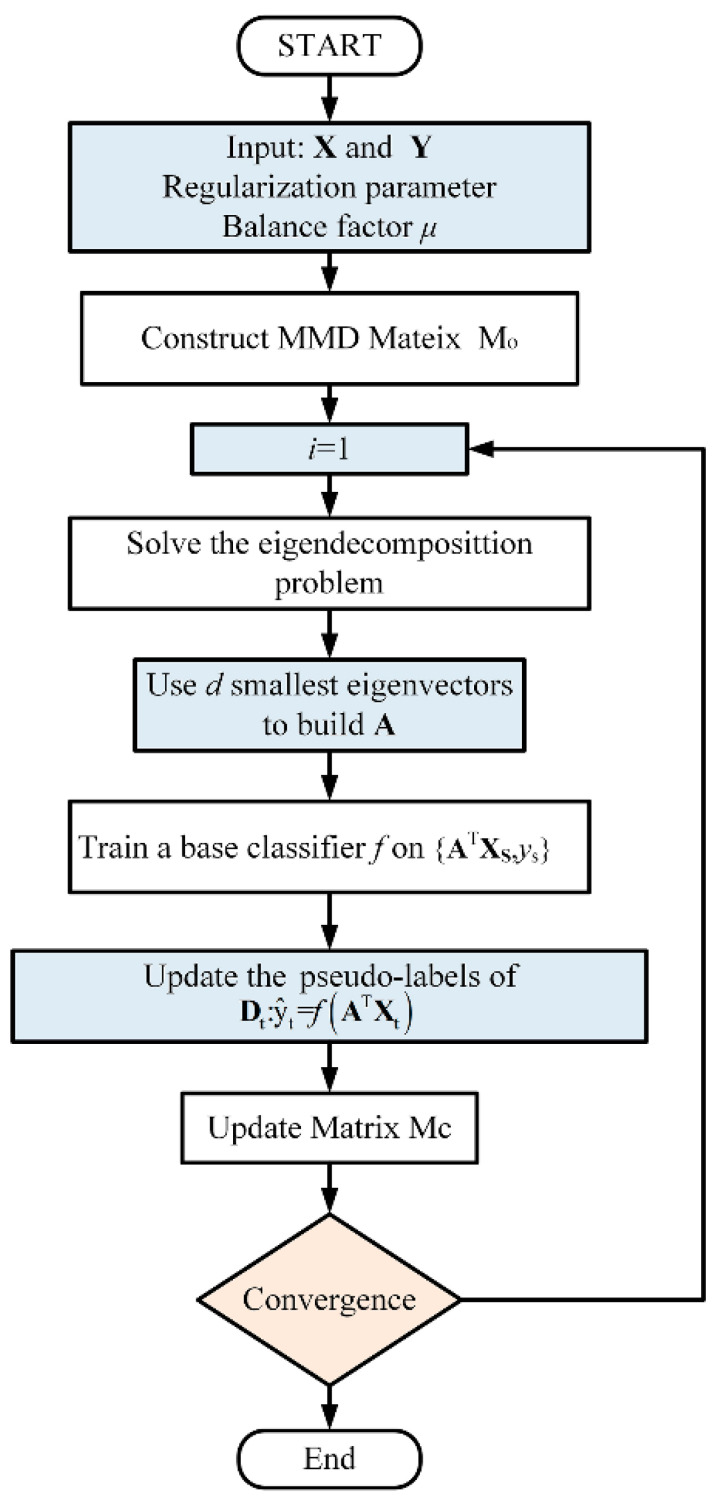
Flowchart of BDA algorithm.

Figure 3 shows the specific details of the BDA algorithm flow.

### 2.3. K-Nearest Neighbors

KNN is a widely used classifier. An advantage of this estimator is that it does not require hyperparameter optimization [23]. There are many researchers that use KNN as a classifier in transfer learning, especially domain adaptation [24,25,26]. KNN is trained on the labeled source data, and tested on the unlabeled target data. Specifically, in the feature space, among the K samples that are most similar (nearest neighbors) to a sample, if most samples belong to a certain category, then a certain sample also belongs to this category. KNN is a classifier that is very sensitive to distance. The most similar K samples are found by calculating the Euclidean distance between the unknown sample and the training set sample.

### 2.4. Particle Swarm Optimization of BDA Parameters

The BDA method was the first to give quantitative estimates of marginal distribution and conditional distribution. However, due to the randomness of MOS sensor drift, it is impossible to directly obtain the appropriate weight balance factor parameter *μ* when constructing the model, so the optimal results cannot be obtained between each set of data. The parameter optimization method is needed to obtain the optimal balance factor parameter of each data set. The weight balance factor parameter *μ* is a single parameter, so the PSO algorithm for parameter optimization can achieve the optimal parameter value.

Suppose a flock of birds conduct a random search for food in an area. There is only one piece of food in this area, and all birds cannot get the food location, but they can judge the distance to the food. In order to find food effectively, the individuals closest to the food in the group search for food. The PSO algorithm is inspired by the group behavior of such organisms to achieve the optimal solution.

If we use particles instead of birds to describe this process, each particle in the model is an individual in the N-dimensional space to search in the space. The current position of each particle in the particle swarm can be regarded as a candidate solution of the current optimization problem. Particles only have two attributes: speed and position. The optimal solution of a single particle is the individual extreme point, and the optimal individual extreme point in the particle swarm is the global optimal solution. After continuous iteration, the position and velocity of the particles are continuously updated until the final convergence condition is met, and the optimal solution is obtained.

The PSO algorithm process for the BDA weight balance factor parameters *μ* in each data set is as follows:

1. Initialization: first, set the number of iterations, the size of the particle swarm, and the position and speed range of the particle swarm. Initialize the initial velocity and position of each particle randomly in the velocity space and the search space. The fitness function is selected as the BDA model.
2. Initial original optimal solution: solve individual extreme points for each particle initially randomly, and obtain a global optimal solution from it, which is recorded as a single global optimal solution.
3. Update speed and position: According to Equations (9) and (10), update the speed and position of the next iteration.
  (9) $$V_{id}=\omega V_{id}+C_{1}random\left(0,1\right)\left(P_{id}-X_{id}\right)+C_{2}random\left(0,1\right)\left(P_{gd}-X_{id}\right)$$
  (10) $$X_{id}=X_{id}+V_{id}$$
  where *w* (*w* > 0) is the inertia factor. The value of *w* represents the strength of the system’s global and local optimization capabilities. *C*_1_ and *C*_2_ are self-learning factors and group-learning factors, respectively, and generally take $C_{1}=C_{2}\in \left[0,4\right]$. *P_id_* represents the *d*-th dimension of the individual extreme value of the *i*-th variable. *P_gd_* represents the *d*-th dimension of the global optimal solution.
4. Iteration termination: when the set number of iterations is reached or within the allowable error range.

## 3. Drift Compensation Methodology and Data Set

### 3.1. Data Set for Validation

We utilized the classification accuracy to verify the drift compensation method proposed for the MOS gas sensor array on the public gas sensor array drift data set [13,27]. This archive is published by the UCI Machine Learning Repository. They used 16 screen-printed, commercially available metal-oxide semiconductor gas sensors for their array, manufactured and commercialized by Figaro Inc. for experiments. At present, it has been widely used in the research of drift compensation of gas sensors.

This data set contains 13,910 measurements from 16 chemical sensors for 36 months utilized in simulations for drift compensation. Six different concentrations of gases collected in the data set are labeled with numbers 1–6, corresponding to ethanol, ethylene, ammonia, acetaldehyde, acetic acid, and toluene, respectively. For ease of processing, the data set was divided into 10 batches. Table 1 summarizes the number of measurements for each class and month included in each batch.

For each sensor, the output response of the sensor was recorded in the form of resistance. The measurement process contained a total of eight features, including two static features, three rising dynamic features, and three decaying dynamic features. As the sensor array, the 16 sensors were divided into four types and four of each. The 8 features extracted from each specific sensor were multiplied by the 16 sensors considered here. A total of 8 × 16 = 128 of the above features were formed into a feature vector dimension. It is worth noting that the 8 features obtained by the same sensor were highly correlated. Meanwhile, the output responses of the four sensors in the same type were also highly correlated. In addition, due to the poor selectivity of MOS sensors, the output of different types of sensors could still have good correlation. This means that the components in the feature vector dimension were highly correlated. This can effectively avoid the so-called dimensional disaster, in which the expected sum converges to a constant due to the existence of a large number of independent components in the data.

**Figure 4:**
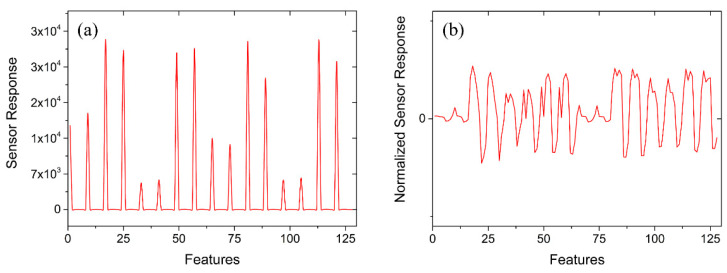
Original sensor array response and normalized sensor array response. (**a**) Original sensor array output response. (**b**) Normalized sensor array output response.

At the same time, the KNN classifier needs to calculate the distance between the samples. If the range of the feature value range is large, the distance calculation is mainly based on the feature, which does not match the actual situation (sometimes, when the range is small, the actual situation is more important). Since the evaluation range of each feature of the sensor in the dataset was inconsistent, it was necessary to normalize the original data, as shown in Figure 4.

### 3.2. Drift Compensation BDA Method Methodology

The main focus of this work is to establish a drift compensation model for the MOS sensor array, combining the BDA method and PSO to solve the drift problem of the electronic nose.

**Figure 5:**
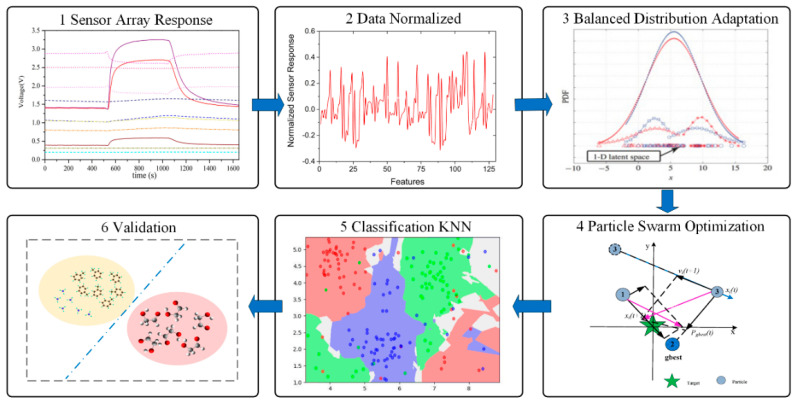
Steps of the BDA developed drift compensation methodology.

The proposed drift compensation model of the MOS sensor array included six parts, as shown in Figure 5. Generally, drift compensation starts with the data collection from sensor arrays. In order to facilitate comparison with other machine learning compensation methods, this study used a public MOS sensor array data set. The 8 features in the sensor response at each measurement point were retained. Since the evaluation of each characteristic index was different, in order to ensure the reliability of the results, it was necessary to normalize the original data, then to apply the BDA method for feature extraction. Because of the adjustment effect of the weight balance factor, a smaller MMD distance and higher accuracy could be achieved than when using JDA and TCA. The reduction of the distribution difference between the two domains based on the data could further improve the robustness of the drift compensation model. The next step was to use the PSO algorithm to optimize the parameters to obtain the optimal weight balance factor. The calculation of the conditional probability distribution needed to know the label of the data. However, the target domain data in the scenario assumed by the BDA drift compensation method was unlabeled. One method was proposed, which was to train a nearest neighbor classifier algorithm in the source domain data, use the nearest-neighbor classifier algorithm to pseudo-label the target domain data, and then use the pseudo-label to calculate the conditional probability distribution of the target domain data. Then, the pseudo-labels were updated through multiple iterations, and the accuracy of the pseudo-labels was gradually improved, thereby improving the performance of the algorithm.

## 4. Experiment and Result Analysis

### 4.1. Experimental Settings and BDA Parameters Configuration

In order to effectively verify the proposed method and facilitate comparison, two experimental settings are given according to [20].

**Setting** **1:** Set batch 1 (source domain) as a fixed training set and tested on batch *K*, *K* = 2,…, 10 (target domains);
**Setting** **2:** The training set (source domain) is dynamically changed with batch *K-1* and tested on batch *K* (target domain), *K* = 2,…, 10.

The number of measurements and classes of test data contained in each batch are summarized in Table 1.

In this study, the accuracy rate of the sample classification of the target domain was used as the basic evaluation criterion of the algorithm effect, and the specific calculation was as follows:

(11) $$Accuracy=\frac{x:x\in D_{t}\wedge f(x)=y(x)}{x:x\in D_{t}}$$

where *f*(*x*) is the true label of the test sample *x*, and *y*(*x*) is the predicted label of the sample *x*.

Five parameters were included in the optimization model of the drift compensation algorithm in this study. Namely, the weight balance factor *μ,* the regularization parameter *λ,* the subspace bases *d,* the number of iterations *T,* and the gamma parameter *γ*.

*λ* is a regularization parameter to ensure that the optimization problem is well-defined. In theory, *λ* controls the complexity of the classification model. When *λ* → 0, the classification model degenerates and leads to serious overfitting problems. When *λ* → ∞, the classification model is too simple to fully fit the discriminative structure of the data.

The subspace bases *d* represents the size used to construct the transformation matrix **A**. *d* is usually chosen as the dimension that makes the subspace sufficiently accurate. The value of *d* cannot exceed the number of features.

For the number of iterations *T*, the BDA method continuously improves the classification accuracy by iteratively updating the pseudo-labels.

The gamma parameter defines the reciprocal of the standard deviation of the RBF kernel. For nonlinear problems, we can use kernel mapping and kernel matrix. Two types of kernel functions, linear kernel $k(x_{i},x_{j})=x_{i}^{T}x_{j}$ and RBF kernel $k(x_{i},x_{j})=e^{-\gamma \|x_{i}x_{j}{\|}^{2}}$, were used in this study.

The parameters *d*, *λ*, and *γ* obtain the best values through deviation optimization. The setting of the number of iterations *T* refers to [22]. Finally, the set of parameter settings was: *d* = 100, *λ* = 1, *γ* = 1, and *T* = 10, respectively.

**Figure 6:**
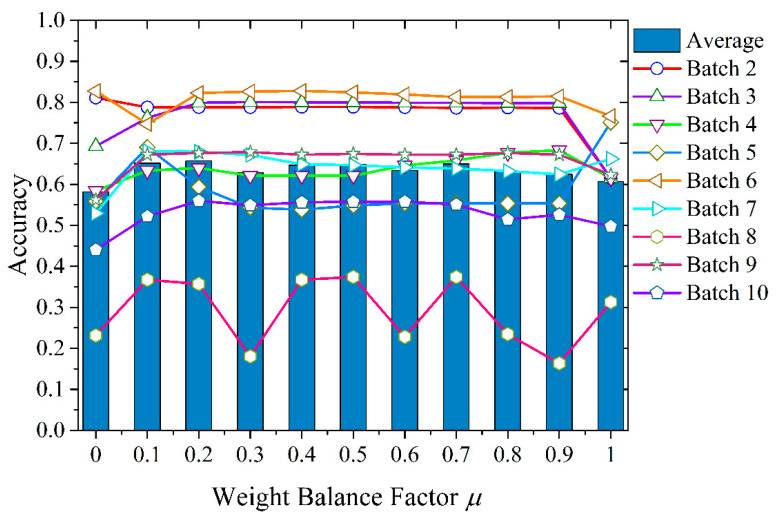
The impact of different weight balance factors *μ* on classification accuracy.

In order to analyze the impact of *μ* on the performance of the BDA method, first, the factor *μ* can be simply regarded as a parameter in the transfer process. In the interval [0, 1], the value of *μ* is taken from 0, and every time 0.1 increases, a set [0, 0.1,…, 1.0] is obtained. Then, an impression of the influence of the value of the weight balance factor *μ* on the result can be obtained, and the RBF kernel function is applied. The experimental results are shown in Table 2 and Figure 6.

Obviously, the optimal value of weight balance factor *μ* varied in different data batches. This showed the importance of marginal and conditional distribution differences across domains. In each batch of data used in the test, the *μ* value corresponding to the best accuracy had no obvious law. Some batches dominated the conditional distribution (for example, Batch 4), and some batches tended to have a certain ratio of the joint distribution of the two (for example, Batch 8).

In order to get the best results, the PSO algorithm was combined to search for the best *µ* of each set of data in a wide range. The parameters in the PSO algorithm were set as follows: the population size was 20; the maximum number of iterations was 10; the weight balance factor optimization limit range was 0 to 1, and the two values of 0 and 1 were considered in advance; the speed limit range was −0.3 to 0.3; and the inertia weight was 0.8.

### 4.2. Performance Verification

**Figure 7:**
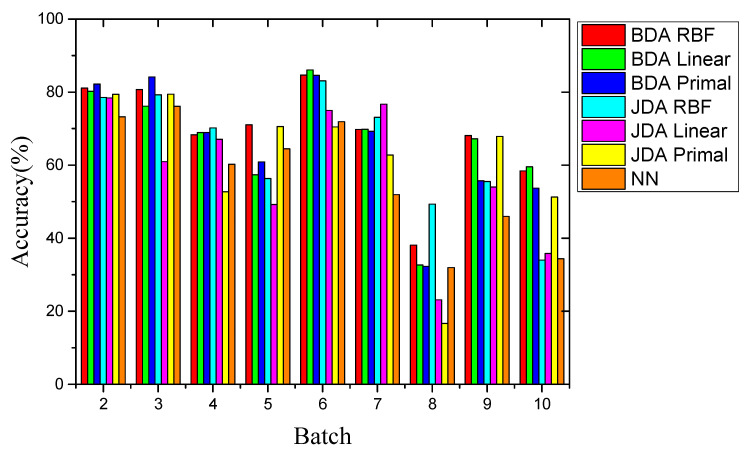
Comparison of recognition accuracy of different methods for Setting 1.

The experiment followed Setting 1 and Setting 2 in turn. We implemented the proposed BDA drift compensation method, which included the primal BDA method, the RBF kernel, and the linear kernel. Table 3 and Table 4 show the best *μ* value and the corresponding best accuracy of each batch of data after the PSO process. The factor *μ* retained three decimal places and was compared with the JDA algorithm with the same kernel. In addition, a non-domain adaptive algorithm using NN as the standard classifier was added. The above drift compensation method based on machine learning using the same dataset is given in [20]. Table 5 shows the accuracy of different methods for 9 batches under experimental Setting 1. Figure 7 intuitively shows the recognition accuracy of all methods.

First, the overall recognition accuracy of the BDA method was higher than that of the comparison method. The BDA method of the RBF kernel optimized by the POS process had the highest average recognition accuracy of 68.92%. Second, compared with the best comparison method JDA, the recognition accuracy was increased by 4.54%, considering that JDA can only adjust marginal and conditional distributions with equal weights (*μ* = 0.5). However, BDA can significantly improve accuracy by adjusting the weight balance parameter *μ* to adapt to different situations. Last, due to the huge distribution gap between the drift data sets, the non-transfer learning method NN only achieved an average recognition accuracy of 56.69%. This showed that the performance of domain adaptation methods was better than that of non-domain adaptation methods. This showed the effectiveness of the transfer learning method, and BDA had the best performance among the three.

**Figure 8:**
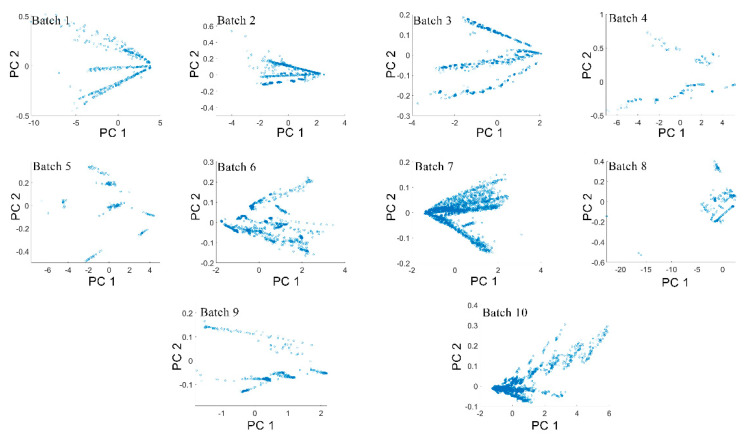
Distribution of principal components of 10 batches.

According to the results, the gas recognition accuracy of the BDA method for Batches 8 and 10 showed the lowest performances, especially for Batch 8, the accuracy of which was only 38.10%. The same was true for other comparison methods, whose performance was much lower than other batches. After long-term operation of the MOS sensor, due to the deterioration of MOS-sensitive materials, the pollution of the MOS gas sensor unit, and the deterioration of the interface electrical contact, the sensor data for Batches 8, 9, and 10 may have been seriously deteriorated. In order to analyze the validity of the data, we applied principal component analysis (PCA) to this data set, and then projected the data into a 2D subspace based on the first two PCs. As shown in Figure 8, the data space distribution of Batch 8 had significant changes compared with other batches. Zhang et al. [14] believed that these changes were caused by drift over time. However, the data space distributions of Batch 9 and 10 were similar to the other batches. Therefore, we believe that the changes in Batch 8 may not have been entirely caused by the drift. Another possible reason was that Batch 8 had a smaller amount of data, and only contained 294 measurement samples. Their combined influence resulted in a huge difference in the distribution for Batch 8.

**Figure 9:**
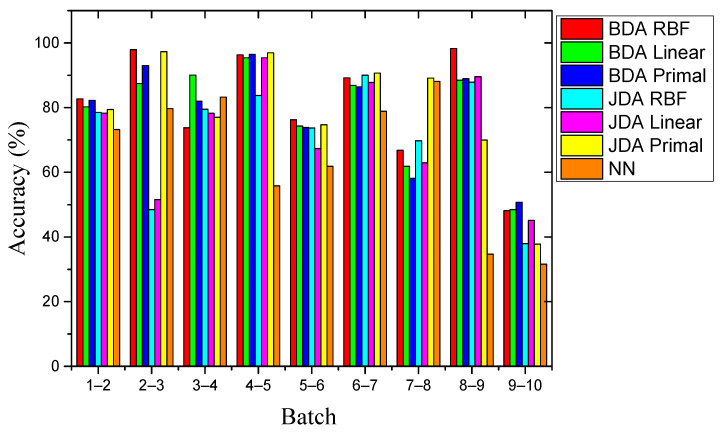
Comparison of recognition accuracy of different methods for Setting 2.

As shown in Figure 9, the performance of all methods for Setting 2 was better than for Setting 1. The possible reason was that the drift influences between batches in Setting 2 were smaller than those in Setting 1, which resulted in a small difference in classification and recognition tasks. The best performance was still the RBF kernel BDA method, with an average recognition accuracy of 81.06%. It is worth noting that the optimal factor *μ* of the RBF kernel BDA in multiple batches was closer to 1, which showed that the conditional distribution was dominant among them. The drift data set of adjacent batches may be more similar. In Batch 2 **→** 3, Batch 4 **→** 5, and Batch 8 **→** 9, the recognition accuracy reached 97.95%, 96.36%, and 98.28%, respectively. In addition, as in Setting1, the recognition accuracy based on the transfer learning method was still much higher than the non-transfer learning NN method. However, in the last batch, the accuracy dropped drastically. We believe that this phenomenon was related to the data collection work of the MOS sensor array. The last collection of sensor array data occurred five months after the previous time. During this time, the sensors were kept powered off. Due to the lack of normal operating temperature, external contaminants may have adhered to the sensitive material layer of sensors. This process is usually irreversible, and sensors were contaminated. This maximized the difference in data distribution between Batch 9 and Batch 10. Although the data in Batch 10 was severely disturbed due to these reasons, the BDA method we proposed was still better than the comparison method when tested for Setting 2. The detailed values of the recognition accuracy of each batch for Setting 2 are recorded in Table 6.

Meanwhile, for Setting 1 and Setting 2 of the drift data of the MOS gas sensor array on 10 different batches, the high-precision results obtained with these two different settings also proved the good robustness of the BDA drift compensation method.

## 5. Conclusions

In this paper, a BDA-based transfer learning method to solve the drift problem of MOS gas sensor arrays was proposed. Specifically, cross-domain feature extraction was performed using the BDA method, and the BDA method simultaneously minimized the difference between the marginal and conditional distributions between domains through the weight balance factor. The PSO algorithm was used to find the best weight balance factor between different data sets to achieve the best classification and recognition accuracy. Experiments on the long-term sensor drift data set collected by the MOS sensor arrays clearly demonstrated the effectiveness of our proposed compensation method. In two different setting groups, the optimized BDA recognition accuracies were 4.54% and 1.62% ahead of the JDA method, and 12.23% and 15.83% ahead of the KNN method. Experiments showed that BDA was significantly better than the JDA domain adaptive method and the KNN basic classifier method in terms of drift recognition accuracy.

In future work, we will further explore and enhance the accuracy of drift compensation to realize real-time drift regression compensation.

## Figures and Tables

**Table 1:** Experimental data of sensor drift in MOS gas sensor array.

Batch ID	Month	1 Ethanol	2 Ethylene	3 Ammonia	4 Acetaldehyde	5 Acetone	6 Toluene
Batch 1	1, 2	83	30	70	98	90	74
Batch 2	3~10	100	109	532	334	164	5
Batch 3	11~13	216	240	275	490	365	0
Batch 4	14, 15	12	30	12	43	64	0
Batch 5	16	20	46	63	40	28	0
Batch 6	17~20	110	29	606	574	514	467
Batch 7	21	360	744	630	662	649	568
Batch 8	22, 23	40	33	143	30	30	18
Batch 9	24, 30	100	75	78	55	61	101
Batch 10	36	600	600	600	600	600	600

**Table 2:** The relationship between weighting factor and precision.

Factor *μ*	0	0.1	0.2	0.3	0.4	0.5	0.6	0.7	0.8	0.9	1.0
Batch 2	81.11	78.78	78.78	78.78	78.86	78.86	78.78	78.62	78.70	78.62	61.49
Batch 3	69.29	76.23	79.89	80.08	80.01	80.01	79.89	79.89	79.82	79.76	61.49
Batch 4	58.39	63.35	63.98	62.11	62.11	62.11	64.60	65.84	67.70	68.32	61.49
Batch 5	55.84	69.04	59.39	54.31	53.81	54.82	55.33	55.33	55.33	55.33	75.13
Batch 6	82.74	74.70	82.26	82.61	82.78	82.43	81.91	81.30	81.30	81.48	76.65
Batch 7	52.92	68.09	67.92	67.17	64.90	64.68	64.13	63.85	63.22	62.44	66.21
Batch 8	23.13	36.73	35.71	18.03	36.73	37.41	22.79	37.41	23.47	16.33	31.29
Batch 9	56.17	67.23	67.66	67.87	67.23	67.45	67.23	67.23	67.66	67.23	62.34
Batch 10	44.06	52.19	55.97	54.89	55.61	55.75	55.78	55.06	51.44	52.58	49.78
Average	58.18	65.15	65.73	62.87	64.67	64.84	63.38	64.95	63.18	62.45	60.65

**Table 3:** The optimal weight balance factor and accuracy of the BDA drift compensation method for Setting 1.

Method	BDA RBF		BDA Linear		BDA Primal	
	*μ*	Acc	*μ*	Acc	*μ*	Acc
Batch 2	0.000	81.11	0.000	80.23	0.012	82.23
Batch 3	0.251	80.71	0.928	76.17	0.256	84.17
Batch 4	0.931	68.32	0.842	68.94	0.658	68.94
Batch 5	0.134	71.07	0.078	57.36	0.367	60.91
Batch 6	0.912	84.65	0.033	86.61	0.148	84.61
Batch 7	0.084	69.78	0.917	69.80	0.803	69.28
Batch 8	0.533	38.10	0.297	32.65	0.700	32.31
Batch 9	0.188	68.09	0.700	67.23	0.907	55.74
Batch 10	0.690	58.44	0.651	59.56	0.961	53.72
Average	68.92		66.45		65.77	

**Table 4:** The optimal weight balance factor and accuracy of the BDA drift compensation method for Setting 2.

Method	BDA RBF		BDA Linear		BDA Primal	
	*μ*	Acc	*μ*	Acc	*μ*	Acc
Batch 1 **→** 2	0.000	82.72	0.000	80.23	0.012	82.24
Batch 2 **→** 3	0.387	97.95	0.762	87.45	0.068	93.00
Batch 3 **→** 4	0.785	73.83	0.683	90.06	0.700	81.99
Batch 4 **→** 5	0.914	96.36	0.848	95.43	0.462	96.45
Batch 5 **→** 6	0.903	76.25	0.537	74.30	0.798	73.87
Batch 6 **→** 7	0.922	89.19	0.474	86.91	0.997	86.41
Batch 7 **→** 8	0.888	66.85	0.730	61.90	0.000	58.16
Batch 8 **→** 9	0.966	98.28	0.055	88.51	0.551	88.94
Batch 9 **→** 10	0.001	48.15	0.892	48.50	0.509	50.72
Average	81.06		79.26		79.09	

**Table 5:** Drift compensation results of BDA and other methods for Setting 1.

Method	Batch 2	Batch 3	Batch 4	Batch 5	Batch 6	Batch 7	Batch 8	Batch 9	Batch 10	Average
BDA RBF	81.11	80.71	68.32	**71.07**	84.65	69.78	38.10	**68.09**	58.44	**68.92**
BDA Linear	80.23	76.17	68.94	57.36	**86.61**	69.80	32.65	67.23	**59.56**	66.45
BDA Primal	**82.23**	**84.17**	68.94	60.91	84.61	69.28	32.31	55.74	53.72	65.77
JDA RBF	78.54	79.26	**70.19**	56.35	83.09	73.12	**49.32**	55.53	34.00	64.38
JDA Linear	78.38	60.97	67.08	49.24	75.00	**76.67**	23.13	54.04	35.83	57.82
JDA Primal	79.42	79.45	62.73	70.56	70.48	62.75	16.67	67.87	51.28	62.36
NN	73.23	76.10	60.25	64.47	71.91	51.95	31.97	45.96	34.39	56.69

**Table 6:** Drift compensation results of BDA and other methods for Setting 2.

Method	1 → 2	2 → 3	3 → 4	4 → 5	5 → 6	6 → 7	7 → 8	8 → 9	9 → 10	Average
BDA RBF	82.72	**97.95**	73.83	96.36	76.25	89.19	66.85	**98.28**	48.15	**81.06**
BDA Linear	80.23	87.45	**90.06**	95.43	74.30	86.91	61.90	88.51	48.50	79.26
BDA Primal	**82.24**	93.00	81.99	96.45	73.87	86.41	58.16	88.94	**50.72**	79.09
JDA RBF	78.54	48.49	79.50	83.76	73.74	90.04	69.73	87.87	37.92	72.18
JDA Linear	78.28	51.60	78.26	95.43	67.35	87.79	62.93	89.57	45.11	75.55
JDA Primal	79.42	97.29	77.02	**96** **.95**	74.70	**90** **.7** **0**	**89** **.12**	70.00	37.80	79.44
NN	73.23	0.79.7	83.23	55.84	61.83	78.88	88.10	34.68	31.58	65.23

## Data Availability

The databases used in this study are public and can be found at the following link: https://archive.ics.uci.edu/ml/datasets/Gas+Sensor+Array+Drift+Dataset+at+Different+Concentrations accessed on 23 October 2013.
